# Supplementary material for: Frequency of Peripheral CD8+ T Cells Expressing Chemo-Attractant Receptors CCR1, 4 and 5 Increases in NPC Patients with EBV Clearance upon Radiotherapy
Source: Cancers (Basel). 2023 Mar 21;15(6):1887. doi: 10.3390/cancers15061887 (PMC10047204; doi:10.3390/cancers15061887)
Supplement: Supplementary file 1 [file cancers-15-01887-s001.zip › supp figures and tables.pdf]

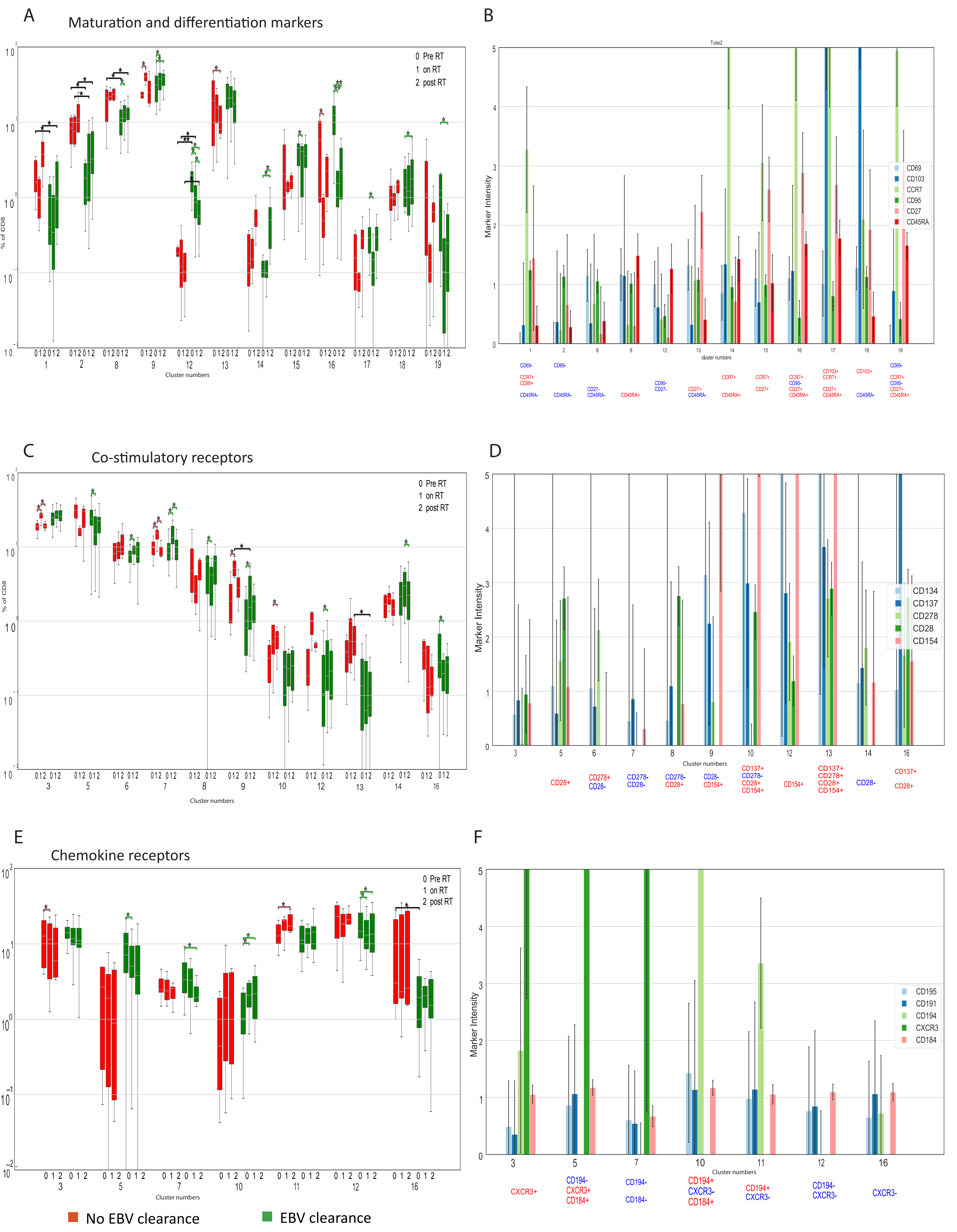

Supplementary figure S1: Clusters of peripheral blood CD8+ T cell subsets in NPC patients with and without post RT plasma EBV DNA clearance.
Analyses of clusters identified by uniform manifold approximation and projection (UMAP) scatter plot of random data points of CD8+ T-cells obtained following flow cytometric staining of PBMCs for expression of (A and B) maturation markers; (C and D) co-stimulatory receptors; and (E and F) chemo-attractant receptors split according to EBV titers and timepoints. Analyses focussed on (A, C and E) differential abundance and (B, D, and F) intensities of such clusters.
Details regarding display of patient groups, time points and statistical analyses are described in legend to Figure 2.

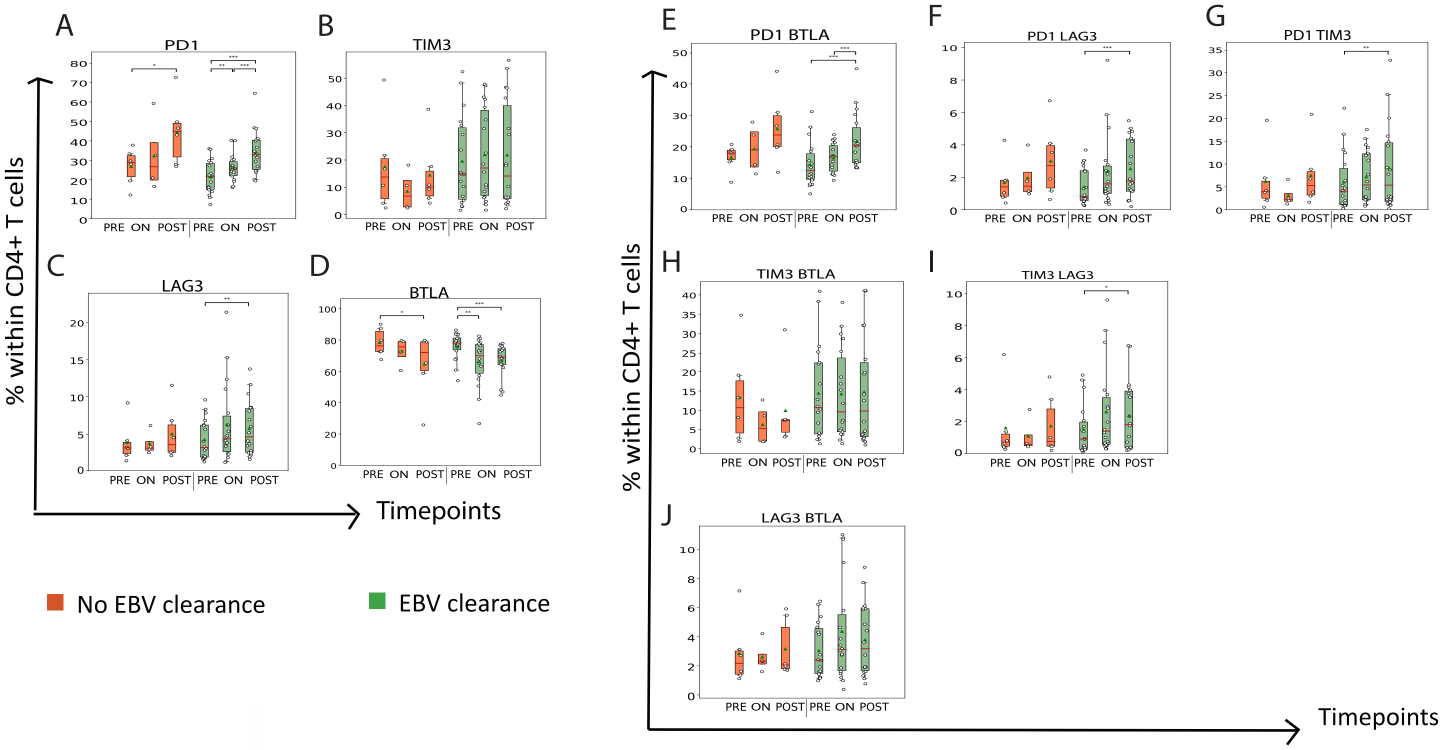

Supplementary figure S2: NPC patients with post RT plasma EBV DNA clearance demonstrate an increase in frequency of LAG3 expressing CD4+ T cells. Boxplots displaying frequency of CD4+ T-cells expressing (A-D) a single type and (E-J) two different types of co-inhibitory receptors. Details regarding display of patient groups, time points and statistical analyses are described in legend to Figure 2.

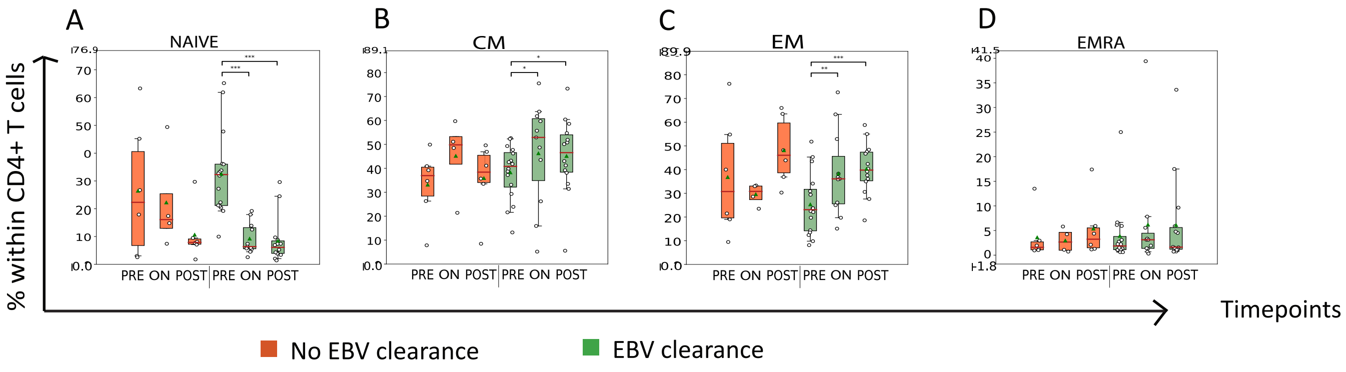

Supplementary figure S3: NPC patients with post RT plasma EBV DNA clearance demonstrate steep drop in frequency of naive CD4+ T cells and increase in frequency of effector memory CD4+ T cells

Boxplots displaying frequency of CD4+ T-cells in different stages of maturation. Details regarding display of patient groups, time points and statistical analyses are described in legend to Figure 2.

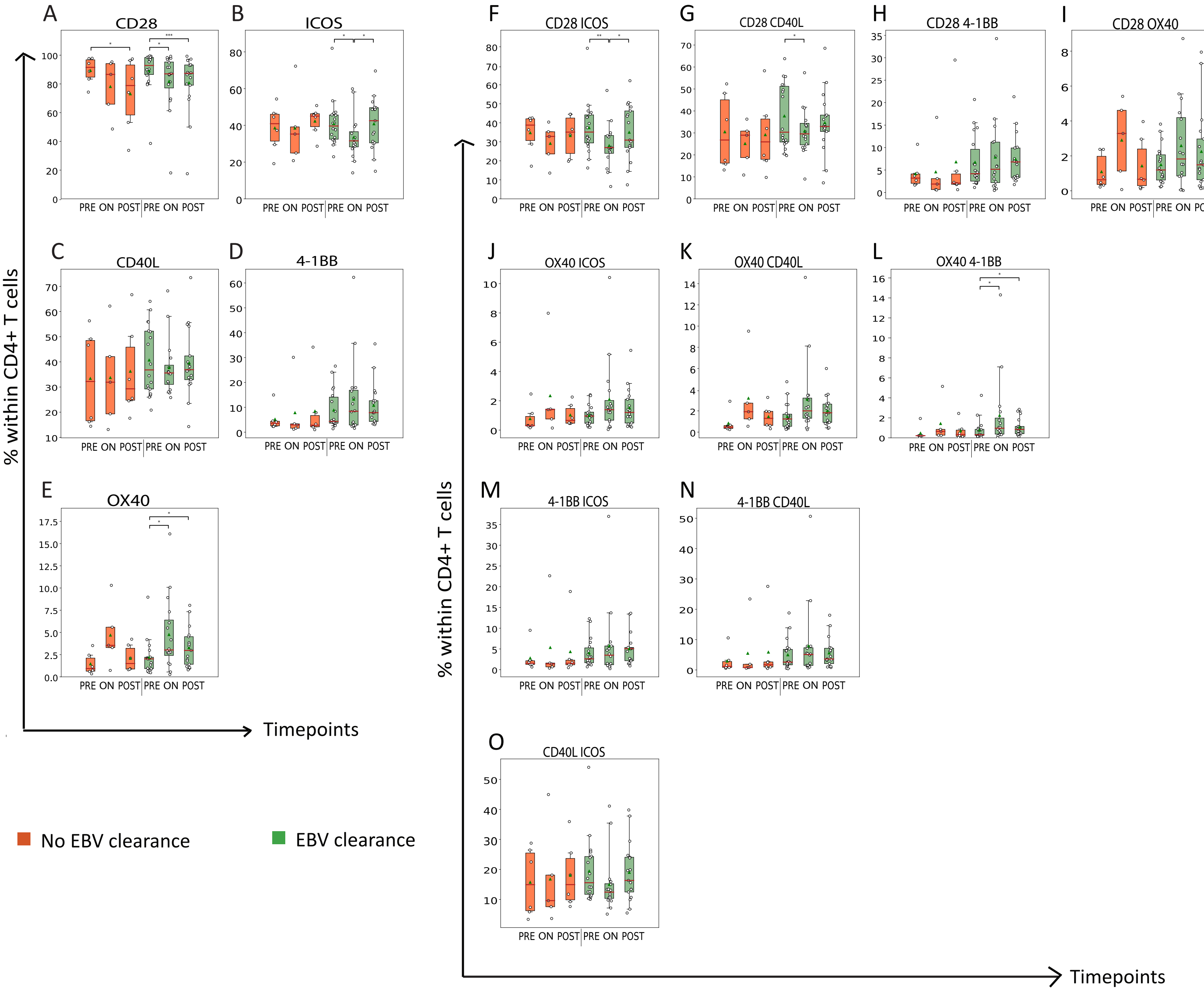

Supplementary figure S4: NPC patients with post RT plasma EBV DNA clearance demonstrate an increase in frequency of OX40 expressing CD4+ T cells. Boxplots displaying frequency of CD4+ T-cells expressing (A) single type and (B) two different types of co-stimulatory receptors. Details regarding display of patient groups, time points and statistical analyses are described in legend to Figure 2.

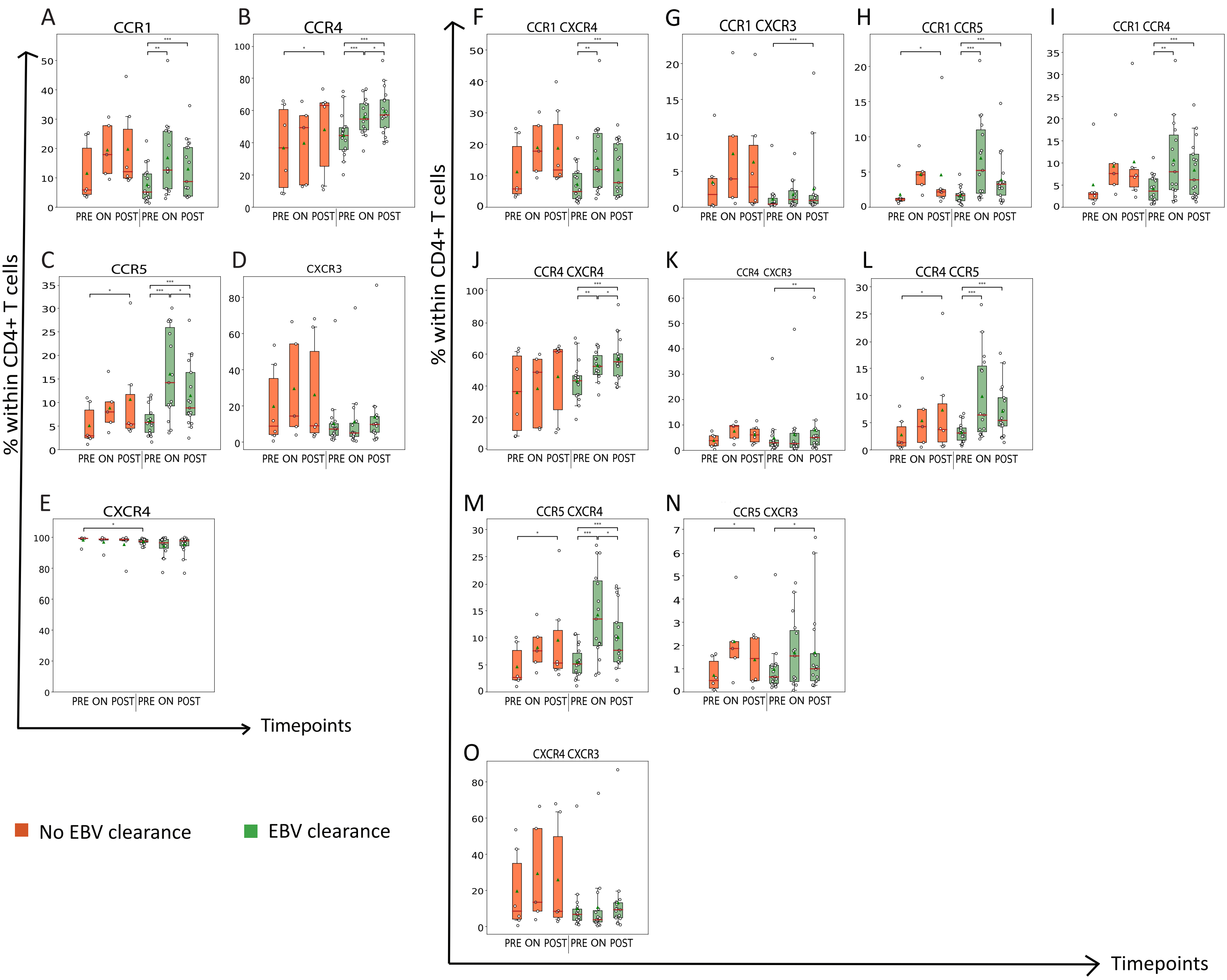

Supplementary figure S5: NPC patients with post RT plasma EBV DNA clearance demonstrate an increase in frequency of chemoattractant receptor CCR1 expressing CD4+ T cells. Boxplots displaying frequency of CD4+ T-cells expressing (A) single type and (B) two different types of chemo-attractant receptors. Details regarding display of patient groups, time points and statistical analyses are described in legend to Figure 2.

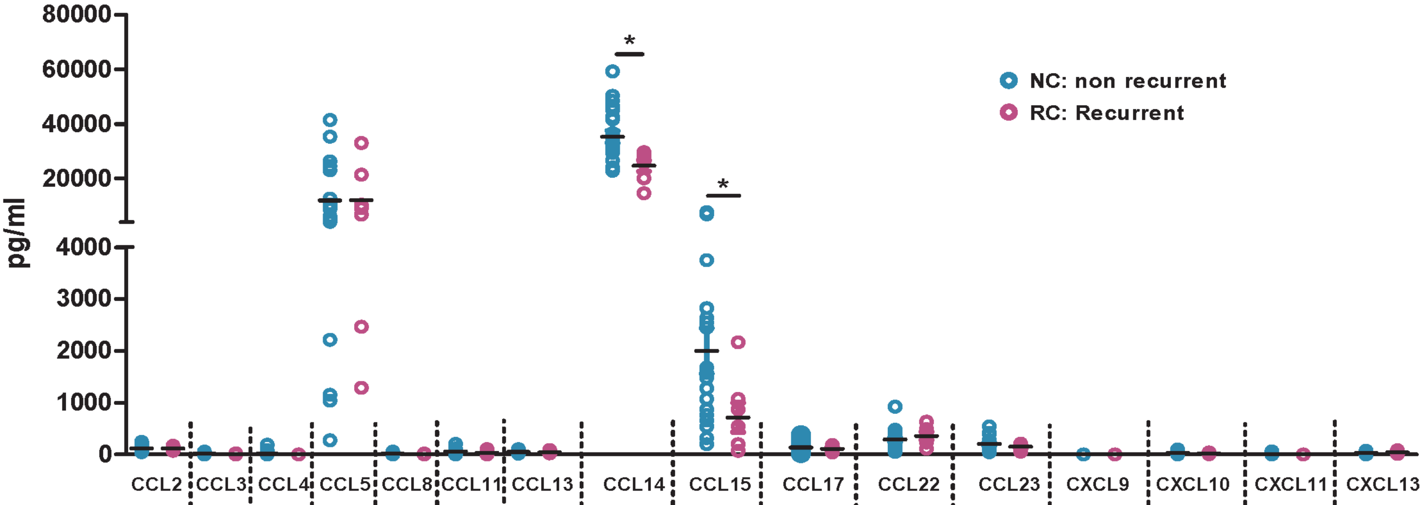

	CCL2	CCL3	CCL4	CCL5	CCL8	CCL11	CCL13	CCL14	CCL15	CCL17	CCL22	CCL23	CXCL9	CXCL10	CXCL11	CXCL13
CCR1	-	L	L	L	L	-	L	L	L	-	-	L	-	-	-	-
CCR4	-	L	-	L	-	-	-	-	-	L	L	-	-	-	-	-
CCR5	L	L	L	L	L	L	L	L	-	-	-	-	-	-	-	-
CXCR3	-	-	-	-	-	-	-	-	-	-	-	-	L	L	L	L

Supplementary figure S6: Higher pre-treatment levels of CCL14 and CCL15 in plasma in non-recurrent NPC patients. Pre-treatment plasma levels for various ligands for CCR1, CCR4, CCR5 and CXCR3 receptor in non-recurrent and recurrent NPC patients and healthy controls. See Materials and Methods for details. Blue: Non recurrent
(n = 20 patients) and pink: recurrent (n = 8)

<table>
<tr><th colspan="3">EBV clearance VS No EBV clearance</th></tr>
<tr><td colspan="2">Pre RT</td><td></td></tr>
<tr><td colspan="2">On RT</td><td>↓ PD1+
CXCR3(CXCR4)</td></tr>
<tr><td colspan="2">Post RT</td><td>↓ PD1+
PD1+LAG-3+</td></tr>
<tr><th colspan="3">Effect of treatment</th></tr>
<tr><td></td><td>No EBV clearance</td><td>EBV clearance</td></tr>
<tr><td rowspan="2">On RT- Pre RT
early changes</td><td rowspan="2"></td><td>↑ Tim-3(LAG3)
CXCR4(CCR1, CCR4, CCR5)
CCR1(CCR4, CCR5)
CCR4(CCR5)</td></tr>
<tr><td>↓ Naïve T
CD28(CD40L, ICOS)</td></tr>
<tr><td rowspan="2">Post RT- Pre RT
late changes</td><td>↑ EMRA T cells</td><td>↑ EM T cells
Tim-3(PD1)
LAG3+BTLA+
OX40(CD40L, ICOS)
CXCR4(CCR1, CCR4)
CCR1(CCR5)</td></tr>
<tr><td>↓ CD28</td><td>↓ Naive T</td></tr>
</table>

Supplementary table S1: **Summary of peripheral CD8+ T-cells subsets with differential frequencies in blood in NPC patients with or without post RT plasma EBV DNA clearance.**

Table summarizing significant differences regarding frequencies of CD8+ T cell subsets between patient groups (plasma EBV DNA clearers vs non-clearers) at different timepoints (pre, on and post-RT) as well as during RT within different patient groups. Statistical analyses were performed using the Mann Whitney U test (between groups) or the Wilcoxen signed rank test (within groups) for T cell subsets according to markers of maturation, co-inhibition, co-stimulation, and chemotaxis. Arrows in upward or downward direction indicate an increase or decrease compared to non-clearers or compared to pre-RT. Underlined markers indicate involvement of a single marker as well as combination of that markers with the other markers mentioned between brackets.

EBV clearance VS No EBV clearance	
**Pre RT**	↓ CXCR4
**On RT**	↓ Naïve T
**Post RT**	

**Effect of treatment**		
	**No EBV clearance**	**EBV clearance**
**On RT- Pre RT**<br>**Early changes**		↑ EM T cells<br>CM T cells<br>PD1<br>OX40(41BB)<br>CXCR4(CCR1, CCR4, CCR5)<br>CCR1(CCR4, CCR5)<br>CCR4(CCR5)
		↓ Naïve T<br>BTLA<br>Tregs<br>CD28(CD40L, ICOS)
**Post RT- Pre RT**<br>**Late changes**		↑ LAG3(PD1, Tim3)<br>PD1(Tim3, BTLA)<br>EM T cells<br>CM T cells<br>OX40(41BB)<br>CXCR4(CCR1, CCR4)<br>CCR1(CCR4, CXCR3)<br>CCR4(CXCR3)
		↓ Naïve T

Supplementary table S2: Summary of peripheral CD4+ T-cells subsets with differential frequencies in blood in NPC patients with or without post RT plasma EBV DNA clearance.
Table summarizing significant differences regarding frequencies of CD4+ T cell subsets between patient groups (clearers vs non-clearers) at different timepoints (pre, on and post-RT) as well as during RT within different patient groups. Statistical analyses were performed using the Mann Whitney U test (between groups) or the Wilcoxen signed rank test (within groups) for T cell subsets according to markers of maturation, co-inhibition, co-stimulation, and chemotaxis. See legend of supplementary table 1 for details.

<table>
<tr><th colspan="3">Non recurrent VS Recurrent</th></tr>
<tr><td colspan="2">Pre RT</td><td></td></tr>
<tr><td colspan="2">On RT</td><td>↓ CXCR3(CXCR4, CCR1, CCR4)</td></tr>
<tr><td colspan="2">Post RT</td><td></td></tr>
<tr><th colspan="3">Effect of treatment</th></tr>
<tr><td></td><td>Recurrent</td><td>Non recurrent</td></tr>
<tr><td rowspan="2">Pre RT– On RT
Early changes</td><td rowspan="2"></td><td>↑ PD1+ Ki67+
Tim3(LAG3)
OX40(CD40L)
CXCR4(CCR1, CCR4, CCR5)
CCR1(CCR4, CCR5)
CCR4(CCR5)</td></tr>
<tr><td>↓ Naïve T
CD28(CD40L, ICOS)
CXCR3(CXCR4)</td></tr>
<tr><td rowspan="2">Pre RT – Post RT
Late changes</td><td rowspan="2"></td><td>↑ EM T cells
EMRA T cells
LAG3(BTLA, PD1, Tim3)
Tim3(PD1)
OX40(CD40L)
CXCR4(CCR1, CCR4)
CCR1(CCR4, CCR5)
CCR4(CCR5)</td></tr>
<tr><td>↓ BTLA
Naïve T
CD28(CD40L)</td></tr>
</table>

Supplementary table S3: Summary of peripheral CD8+ T-cells subsets with differential frequencies in blood in recurrent and non-recurrent NPC patients at different timepoints. Table summarizing significant differences regarding frequencies of CD8+ T cell subsets between patient groups (non-recurrent vs recurrent) at different timepoints (pre, on and post-RT) as well as during RT within different patient groups. Statistical analyses were performed using the Mann Whitney U test (between groups) or the Wilcoxen signed rank test (within groups) for T cell subsets according to markers of maturation, co-inhibition, co-stimulation, and chemotaxis. See legend of supplementary table 1 for details.
